# Improved Blue, Green, and Red Fluorescent Protein Tagging Vectors for *S. cerevisiae*

**DOI:** 10.1371/journal.pone.0067902

**Published:** 2013-07-02

**Authors:** Sidae Lee, Wendell A. Lim, Kurt S. Thorn

**Affiliations:** 1 UCSF Center for Systems and Synthetic Biology, University of California San Francisco, San Francisco, California, United States of America; 2 Department of Cellular and Molecular Pharmacology, University of California San Francisco, San Francisco, California, United States of America; 3 Howard Hughes Medical Institute, University of California San Francisco, San Francisco, California, United States of America; 4 California Institute for Quantitative Biomedical Research, San Francisco, California, United States of America; 5 Department of Biochemistry and Biophysics, University of California San Francisco, San Francisco, California, United States of America; Dartmouth College, United States of America

## Abstract

Fluorescent protein fusions are a powerful tool to monitor the localization and trafficking of proteins. Such studies are particularly easy to carry out in the budding yeast *Saccharomyces cerevisiae* due to the ease with which tags can be introduced into the genome by homologous recombination. However, the available yeast tagging plasmids have not kept pace with the development of new and improved fluorescent proteins. Here, we have constructed yeast optimized versions of 19 different fluorescent proteins and tested them for use as fusion tags in yeast. These include two blue, seven green, and seven red fluorescent proteins, which we have assessed for brightness, photostability and perturbation of tagged proteins. We find that EGFP remains the best performing green fluorescent protein, that TagRFP-T and mRuby2 outperform mCherry as red fluorescent proteins, and that mTagBFP2 can be used as a blue fluorescent protein tag. Together, the new tagging vectors we have constructed provide improved blue and red fluorescent proteins for yeast tagging and three color imaging.

## Introduction

The ability to directly modify the genome of the budding yeast *Saccharomyces cerevisiae* by homologous recombination is a major advantage of this model system. In particular, PCR-based recombination methods allow the targeting of any region in the genome by amplifying a cassette with primers containing short (40 bp) regions homologous to the desired integration site. PCR-based recombination has been used for both deletion of yeast genes and fusion of tags to those genes (reviewed in [1]). Such gene-tagging approaches are particularly powerful as they leave the tagged gene in its native chromosomal context, expressed under its native promoter. Furthermore, in a haploid yeast cell, the tagged gene will be the only copy of that gene present, allowing for easy assessment of whether the tagged gene has a phenotype.

A wide variety of tagging vectors are available for fusing different fluorescent proteins to yeast proteins. These allow imaging of the tagged protein by fluorescence microscopy so that its spatial distribution and transport can be determined. As the tagged gene will be the sole copy present in a haploid cell, this also allows the measurement of protein abundance by fluorescence intensity measurements or by fluorescence correlation spectroscopy [2]. Spectrally separated fluorescent proteins allow multiple tagged proteins to be imaged and protein interactions to be monitored by resonance energy transfer [3]. Tagging vectors are available for constructing gene fusions to a wide variety of fluorescent proteins: green fluorescent protein and its blue, cyan, and yellow variants, red fluorescent proteins, and the photoactivatible proteins PA-GFP and mEos2 [3]–[9].

Recent advances in fluorescent protein engineering have produced many fluorescent proteins with desirable properties. Fluorescent proteins now span a wide range of colors, with bright blue fluorescent proteins complementing green and red fluorescent proteins. Significant improvements in green and red fluorescent protein performance have been described with the generation of brighter, more photostable, and faster maturing fluorescent proteins. However, these newer proteins have not been systematically tested in *S. cerevisiae*, leaving it unclear which of these proteins will perform best in yeast.

Here, we have optimized and systematically tested a number of blue, green, and red fluorescent proteins for use in yeast protein tagging. We have also optimized and tested two long Stokes shift fluorescent proteins, and one far-red protein reported to be fluorescent when excited at 640 nm. We have assessed these proteins for brightness, photostability, and perturbation of fusion proteins, and have recommendations for optimal blue, green, and red fluorescent proteins for imaging tagged proteins in yeast. In particular, we identify red fluorescent proteins that are several-fold brighter than the commonly used mCherry, and a bright blue fluorescent protein for imaging in yeast.

## Materials and Methods

### Plasmid Construction

The plasmid backbones were derived from pFA6a-link-tdimer2-SpHIS5 (pKT146), pFA6a-link-tdimer2-SpURA3 (pKT176), and pFA6a-link-tdimer2-Kan (pKT178) [6]. Protein sequences for fluorescent proteins were taken from the literature and the corresponding DNA sequences were optimized for *S. cerevisiae* expression by DNA2.0 [10]. All protein sequences are shown in Sequences S1 and are identical to the literature sequences except for GFPγ, which contains the additional mutations S72A (known to improve folding) and L231H. The resulting sequences were tailed with PacI and AscI sites and synthesized by DNA2.0. The resulting fluorescent proteins were subcloned into the pFA6a-link backbones using the PacI and AscI sites, replacing tdimer2 with the desired fluorescent protein.

All plasmids are available from Addgene (www.addgene.org) except for TagBFP, TagBFP2, TagRFP-T, TagRFP657, LSS-mKate2, mKate2, and PA-TagRFP. These incorporate sequences sold by Evrogen and cannot be distributed by Addgene.

### Yeast Gene Tagging

Genes were tagged in *S. cerevisiae* strain BY4741 by PCR-mediated transformation [11]. Tagging cassettes were amplified with KOD Hotstart PCR (EMD Millipore) using the forward primer (gene-specific sequence)-GGTGACGGTGCTGGTTTA and the reverse primer (gene-specific sequence)-TCGATGAATTCGAGCTCG. Overnight cultures of *S. cerevisiae* (10 mL) were diluted into 100 mL of fresh media, grown to OD 0.7–1.0, washed twice with 0.1 M lithium acetate/1× TE and resuspended in 2 mL 0.1 M lithium acetate/1× TE. 20 µl of the PCR product was then incubated with 200 µl of washed cells, 10 µl of ssDNA, and 1.2 ml of 0.1 M lithium acetate/1× TE/50% PEG3350 and incubated at 30°C for 30 min. Cells were then heat shocked at 42°C for 15 min after adding 154 µl of DMSO. The cells were then pelleted, resuspended in 100 ul of water and spread on selective media. Tagging of the targeted gene of interest was confirmed by colony PCR to verify the presence of both integration junctions and the absence of the unmodified gene [1].

### Yeast Imaging

For imaging, cells were grown overnight in low fluorescence SC media [6], diluted 1∶20–1∶100 in fresh media and then grown three hours before imaging. Cells were immobilized on concanavalin A-coated glass bottom 35 mm dishes. Widefield microscopy was performed on a Nikon Ti microscope with a Photometrics Coolsnap HQ2 camera, using 60×/1.4 NA or 100×/1.4 NA oil immersion lenses. Illumination was provided by a Lambda XL lamp (Sutter Instrument Company, Novato CA). Chroma fluorescence filter sets 89021 and 89000 were used, with the specific channels as follows: GFP: ET470/20×, ET525/50m; mCherry: ET572/35×, ET632/60m; DAPI: ET402/15×, ET455/50m; Cy3: ET555/25×, ET605/52m; Cy5: ET645/30×, ET705/72m; mKeima: ET402/15×, ET605/52m.

Spinning disk confocal imaging was performed on a Nikon Ti-E equipped with a Yokogawa CSU-22 spinning disk confocal and a Photometrics Evolve EMCCD camera, using a 100×/1.4 NA oil immersion lens. Laser illumination was at 405 nm (BFP), 491 nm (GFP), or 561 nm (RFP), and detection filters were ET460/50m (BFP), ET525/50m (GFP), or ET610/60m (RFP).

Image analysis was performed in NIS-Elements (Nikon Instruments Inc.). The background was estimated for each image from a region free of cells and subtracted. For time lapse images, this subtraction was performed at each time point. For brightness measurements, the image was thresholded to identify cells, and the intensity was calculated for each cell. The mean intensity of all cells from three or more images was recorded (typically 100s of cells). For time lapse photobleaching images, the same procedure was followed at each time point. The time point at which point the mean intensity dropped below 50% was then determined, and the sum of the mean intensity at all prior time points (the integrated intensity) was calculated.

## Results

### Construction of Novel Yeast Fluorescent Protein Tagging Vectors

We set out to systematically test recently developed fluorescent proteins for use in protein tagging in yeast. To do so, we first collected a list of bright fluorescent proteins recently published in the literature, as well as those recommended by our colleagues. This list includes both commercially and academically developed proteins. We focused on proteins compatible with the common four color filter set used for imaging DAPI/FITC/Cy3/Cy5 and with 405 nm, 488 nm, 561 nm, and 640 nm lasers on a confocal microscope. The complete list of proteins tested along with their photophysical properties is provided in Table 1, and the protein sequence of each protein is provided in Sequences S1. Specifically, we chose two blue fluorescent proteins, seven green fluorescent proteins, and seven red fluorescent proteins. We also included one far-red protein, TagRFP657, that is reported to be detectable under 640 nm excitation, and two long-Stokes shift proteins that we hoped might allow five-color imaging by exciting at 405 nm and detecting in the Cy3 emission filter. We additionally constructed, but did not test, five photoactivatible and photoconvertible fluorescent proteins (Table S1).

**Table 1:** Fluorescent Proteins Tested.

Protein	λex	λem	QY	EC	Brightness	Reference
Blue Fluorescent Proteins:						
mTagBFP	402	457	0.63	52000	32.8	[27]
mTagBFP2	399	454	0.64	50600	32.4	[28]
Green Fluorescent Proteins:						
EGFP	488	507	0.6	56000	33.6	[29], [30]
Clover	505	515	0.76	111000	84.4	[16]
Emerald	487	509	0.68	57500	39.1	[15]
GFPγ						[31]
MaxGFP						Amaxa
Superfolder GFP	485	510	0.65	83300	54.1	[32]
mWasabi	493	509	0.80	70000	56.0	[13]
Red Fluorescent Proteins:						
mCherry	587	610	0.22	72000	15.8	[33]
mApple	568	592	0.49	75000	36.8	[20]
mKate2	588	633	0.4	62500	25.0	[21]
mKO2	551	565	0.62	63800	39.6	[34]
mRuby	558	605	0.35	112000	39.2	[35]
mRuby2	559	600	0.38	113000	42.9	[16]
TagRFP-T	555	584	0.41	81000	33.2	[20]
Others:						
TagRFP657	611	657	0.10	3400	0.34	[36]
mKeima	440	620	0.24	14400	3.5	[37]
LSS-mKate2	460	605	0.17	26000	4.4	[38]

λex and λem are the peak excitation and emission wavelengths of the fluorescent protein, respectively. QY is the quantum yield and EC the extinction coefficient in M^−1^ cm^−1^. Brightness is the product of QY and EC, divided by 1000. Data was taken from the literature and is not available for GFPγ or MaxGFP.

**Figure 1:**
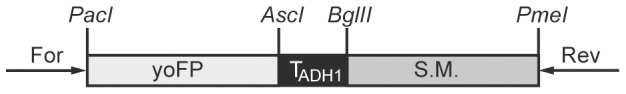
Schematic design of tagging plasmids. The overall design of these plasmids is identical to the pFA6a-link tagging plasmids previously published [6]. yoFP is one of the 24 yeast optimized proteins cloned here and S.M. is the yeast selectable marker, either *SpHis5*, *CaUra3*, or KanR. These tagging sequences can be amplified with the forward primer (gene-specific sequence)-GGTGACGGTGCTGGTTTA and reverse primer (gene-specific sequence)-TCGATGAATTCGAGCTCG. A complete list of plasmids constructed in this study is in Table S2.

Because codon usage has been shown to significantly affect fluorescence intensity of fusion proteins [6], and additional factors such as RNA secondary structure can affect expression level [12], we had each protein optimized by DNA2.0 for expression in *S. cerevisiae*. This ensures that differences in codon usage between proteins do not affect our comparison and that the sequences we are testing are optimized for yeast expression. The resulting yeast optimized fluorescent proteins (denoted by yo followed by the fluorescent protein name; e.g. yoEGFP) were then cloned into the pFA6a-link tagging vectors we have previously published [6]. The proteins were cloned into vectors with the selectable markers CaUra3, SpHis5, and KanR (G418 resistance), giving a set of 72 vectors (Figure 1 and Table S2). These vectors share the same tagging primers (forward: (gene-specific sequence)-GGTGACGGTGCTGGTTTA; reverse: (gene-specific sequence)-TCGATGAATTCGAGCTCG) as our previous vectors and can be used interchangeably with them.

### Fluorescent Protein Brightness

To test the performance of these proteins, we first fused each fluorescent protein to the highly abundant metabolic gene Tdh3. As the resulting fusions are identical except for the sequence of the fluorescent protein, we expect these fusions to accurately reflect the performance of these tags. The brightness of different tags can differ for a number of reasons, including differences in the photophysical properties of the tag (e.g. quantum yield or extinction coefficient) or poor expression or folding of the tag. However, we expect that these properties should be independent of the protein being tagged and so that this should be a reliable reporter of the tag performance in other applications. While all the versions tested are yeast optimized, we have omitted the ‘yo’ prefix below for clarity.

We first measured the relative detectability of each protein by comparing the brightness of tagged cells to that of untagged cells as imaged on a widefield microscope. This is a measure of the signal-to-background ratio (SBR) for each protein. By this metric, the commonly used proteins EGFP and mCherry have SBRs of ∼180. Several of the tested performed very poorly in this assay. The two long-Stokes shift proteins, mKeima and LSS-mKate2 and the far-red fluorescent protein TagRFP657, had SBRs <5 and were not studied further. In the case of the long-Stokes shift proteins this poor performance likely reflects both their low intrinsic brightness and that our filters were poorly matched to their spectra; we excited with light centered at 402 nm and these proteins are optimally excited at 440–460 nm. The poor matching of our filters results from the fact that we were using a four-band filter set optimized for DAPI/FITC/Cy3/Cy5. A filter set designed for imaging CFP/YFP/RFP might perform better with these proteins. The poor performance of TagRFP657 likely results from both low intrinsic brightness and poor matching to filters designed for Cy5; nevertheless, as the longest-wavelength intrinsically fluorescent protein identified to date, we wanted to determine if this protein was bright enough to be useful for yeast imaging. The blue fluorescent protein mTagBFP also had an SBR less than 5. The improved mTagBFP2 is about ten times brighter and is only ∼5-fold less detectable than EGFP, making it a viable tag for imaging with DAPI filters and 405 nm excitation.

**Figure 2:**
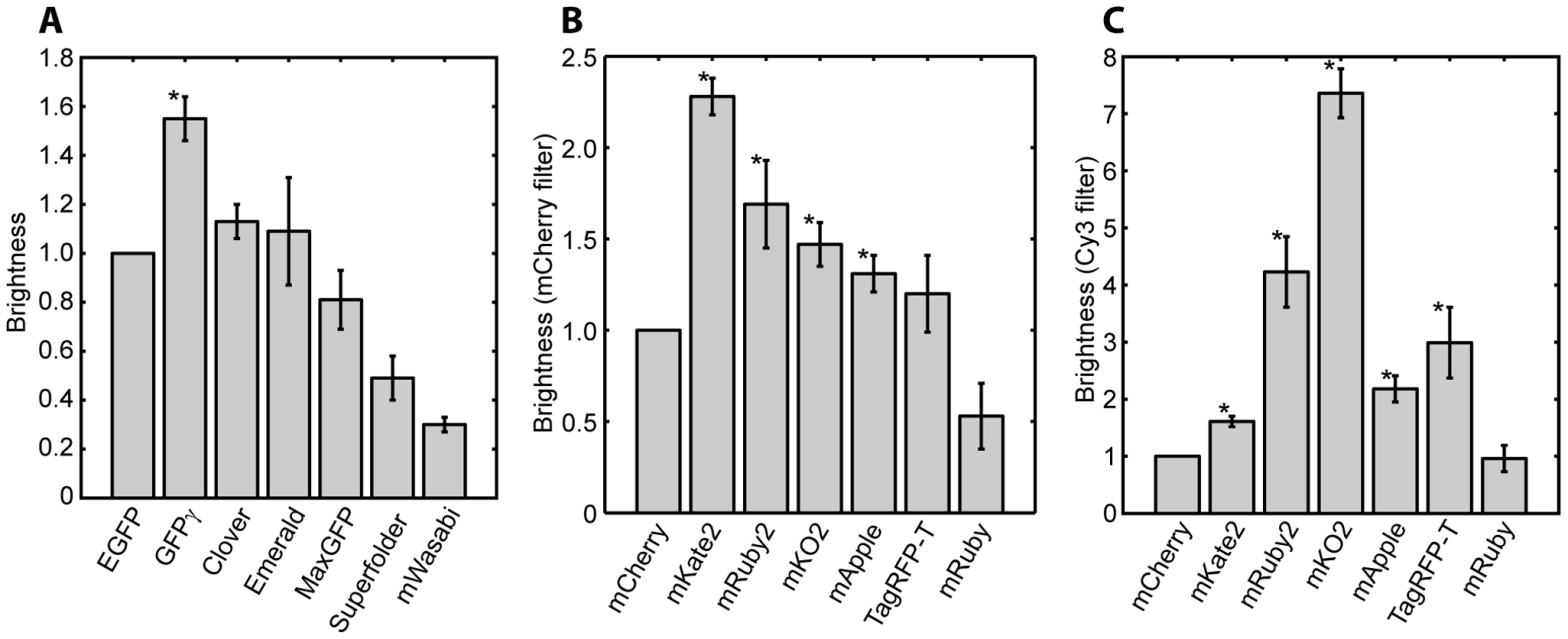
Brightness of green and red fluorescent proteins. Yeast expressing fusions of each of the optimized fluorescent proteins to the TDH3 protein were imaged, and the mean fluorescence of each strain was calculated. Data from each day was normalized to EGFP (for green proteins) or mCherry (red proteins) to compensate for day-to-day fluctuations in lamp brightness and detection efficiency. The measurement was repeated on at least three days and the mean and standard error for each strain is plotted. * indicates a protein significantly brighter than EGFP or mCherry as determined by a one-sided t-test with 5% significance threshold. A. Green fluorescent proteins. B. Red fluorescent proteins imaged with an mCherry filter set. C. Red fluorescent proteins imaged with a Cy3 filter set.

We next systematically compared the multiple green and red fluorescent proteins we had produced. We first assessed their brightness by comparing the relative brightness of each green fluorescent protein to EGFP and each red fluorescent protein to mCherry. Because the red fluorescent proteins have varying excitation spectra we evaluated their brightness using two different commonly used filter sets, one designed for imaging mCherry and one designed for imaging Cy3. The results of this comparison are shown in Figure 2 and Table S3. Strikingly, most of the green fluorescent proteins perform no better than EGFP, with the exception of GFPγ, which is approximately 50% brighter. Despite its dimness, Wasabi may be useful for certain experiments as, unlike other GFPs, it is not excited in the near UV and can be multiplexed with the UV-excited GFP T-Sapphire [13], [14]. The improved EGFP variants Clover and Emerald [15], [16], which are reported to be substantially brighter than EGFP, perform comparably to it under these conditions. These proteins derive their high brightness in part because of optimization for folding at 37°C; it is possible that the mutations conferring improved folding at 37°C in bacteria and mammalian cells as free protein are unimportant for folding at 30°C in yeast as a C-terminal fusion protein. Furthermore, the observed brightness in *S. cerevisiae* is poorly correlated with the photophysical brightness (product of quantum yield and extinction coefficient), suggesting that factors other than the intrinsic chromophore brightness are important for the measured brightness. In addition to protein folding, this could include rapid photobleaching or interactions with the ionic or redox environment in the cell [17]–[19].

We find many red fluorescent proteins that are brighter than mCherry. Furthermore, because of the wide spectral range spanned by red fluorescent proteins, the optimal choice of protein depends on the choice of filter set used to view it. Figures 2B and C and Table S4 show the relative brightness of these seven red fluorescent proteins as measured through a Cy3 filter set and an mCherry filter set. Not surprisingly, the results differ substantially, with mCherry performing much more poorly when imaged with the Cy3 filter set. In both cases, however, we find a number of proteins that outperform mCherry. We also compared the brightness of each protein as measured through the Cy3 filter set with that of mCherry as measured through the mCherry filter set to assess what the best protein imaged through either filter set is (Table S4). For the mCherry filter set, the brightest protein is mKate2, 2.3× brighter than mCherry. For the Cy3 filter set, mRuby2 and mKO2 are 2.3× and 3.1× brighter, respectively, than mCherry in the mCherry filter set. Overall, these are the three brightest red fluorescent proteins.

We also compared the brightness of these green and red proteins when imaged under laser illumination with a spinning disk confocal. Under these conditions, the brightness of the green fluorescent proteins was very similar to that observed in the widefield measurements above (Table S3). For red fluorescent proteins, we see that the proteins which perform well when imaged with the Cy3 cube also perform well when imaged with the spinning disk confocal, although the relative performance improvement compared with mCherry is larger when imaged with the spinning disk confocal (Table S4).

### Fluorescent Protein Stability

Brightness is not the only important parameter when choosing a fluorescent protein. For time lapse imaging, a critical parameter is the photostability of the protein. Photobleaching occurs when a fluorophore in the excited state undergoes a chemical reaction leading to its irreversible destruction. Accordingly, photobleaching limits the amount of data that can be recorded in a timelapse acquisition. To measure the photobleaching rate we captured sequential images of each fluorescent protein tagged to Tdh3 under continuous illumination. We then quantified the time required to bleach to 50% of the initial intensity and the integrated intensity of the cell during this time. This latter measurement is probably the most relevant for assessing the performance of fluorescent proteins as it measures the total amount of photons that can be detected from a fluorescent protein until it drops to half of its initial intensity. It also takes into account the intrinsic brightness of the protein and partially corrects for illumination intensity changes: if the illumination brightness decreases, the brightness decreases but so does the photobleaching rate.

**Figure 3:**
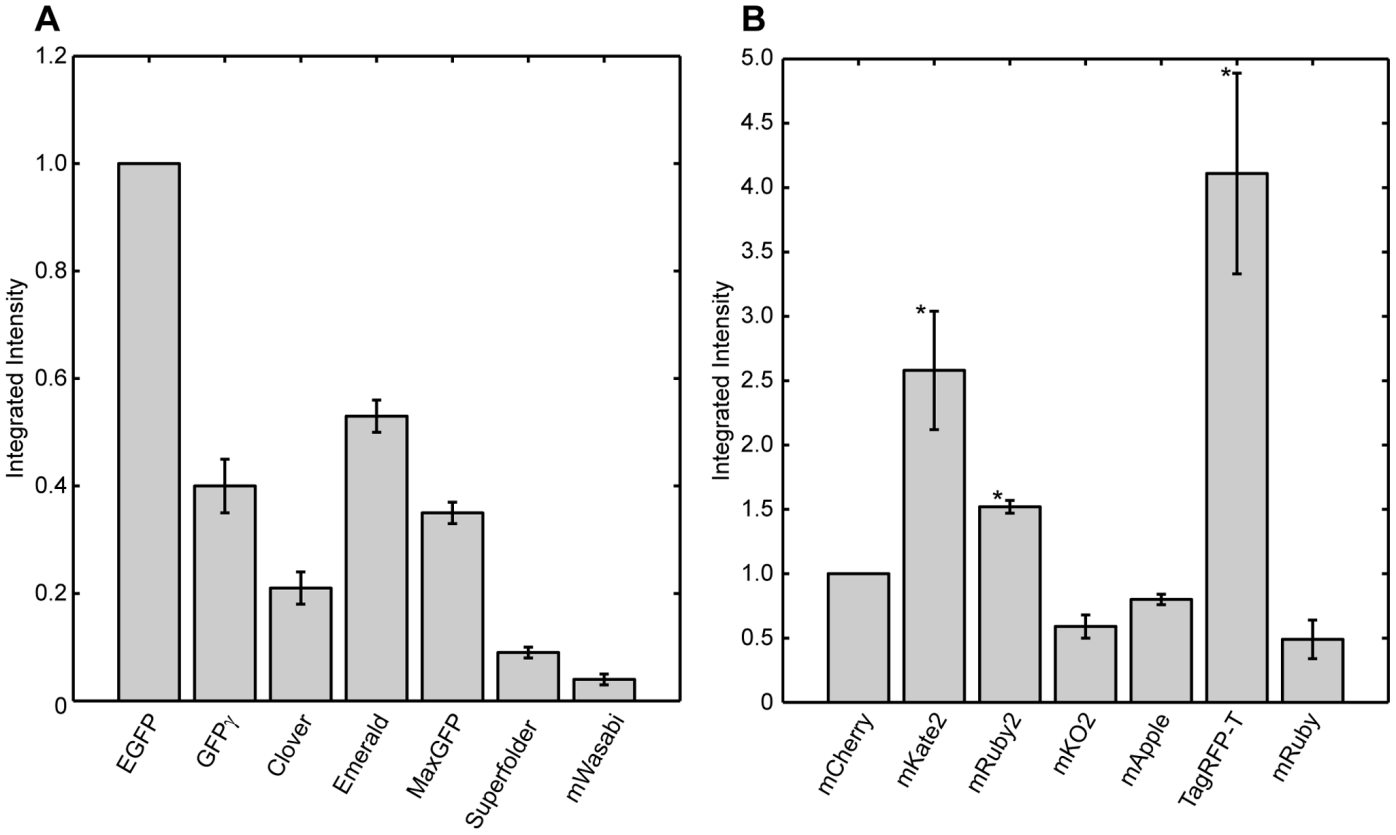
Photostability of red and green fluorescent proteins. Yeast expressing fusions of each of the optimized fluorescent proteins to the TDH3 protein were imaged continuously until their intensity dropped below 50% of the initial intensity. The intensity of each cell integrated over the time until 50% bleaching occurred was then calculated, and the mean integrated intensity for each strain on each day was normalized to EGFP (for green proteins) or mCherry (red proteins) to compensate for day-to-day fluctuations in lamp brightness and detection efficiency. The measurement was repeated on at least two days and the mean and standard error for each strain is plotted. * indicates a protein with significantly larger integrated intensity than mCherry as determined by a one-sided t-test with 5% significance threshold.

The integrated intensities measured during bleaching to 50% of initial intensity for both the green and red fluorescent proteins are shown in Figure 3 and Tables S3 and S5. Surprisingly, none of the green fluorescent proteins tested perform better than EGFP; even those proteins brighter than EGFP are substantially less photostable. For red fluorescent proteins, imaged through the mCherry filter, we find three RFPs, mRuby2, mKate2, and TagRFP-T, that have significantly higher integrated intensities than mCherry. mRuby2 actually bleaches slightly more rapidly than mCherry, but its higher brightness more than compensates for its rapid bleaching. mKate2 is brighter than mCherry, but bleaches at about the same rate, while TagRFP-T is about the same brightness as mCherry but bleaches much more slowly. TagRFP-T was selected for photostability so this is not surprising [20].

### Perturbation of Fusion Protein Function

**Figure 4:**
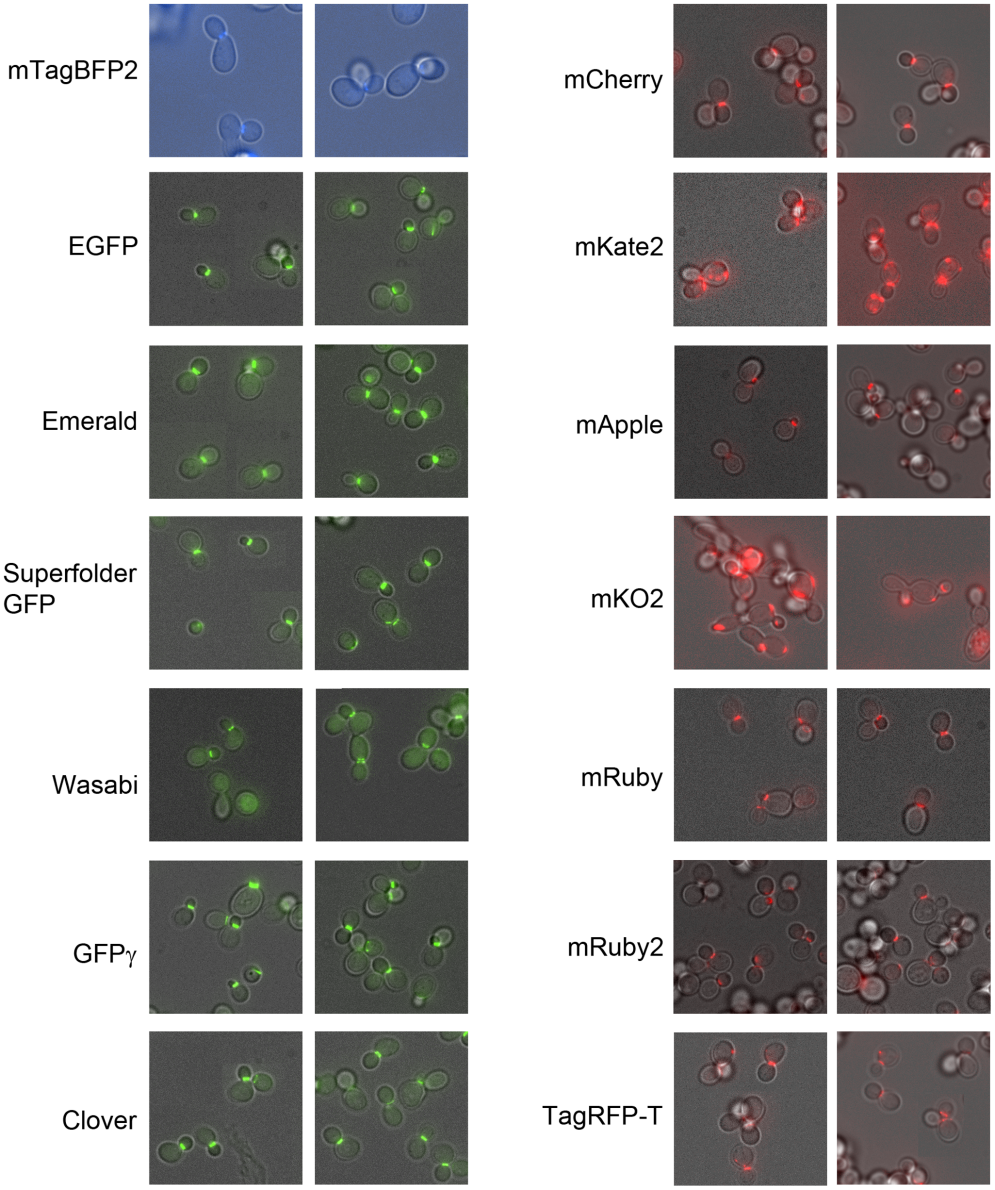
Perturbation of protein function. Yeast expressing fusions of each of the indicated proteins to the C-terminus of Cdc12 were imaged to assess whether they perturb its function. Perturbation of Cdc12 function manifests as misshapen yeast cells and/or mislocalized Cdc12. The green fluorescent proteins show minimal perturbation; mKate2 and mKO2 show major perturbation; mTagBFP2 is intermediate. Brightness has been normalized separately for each image so it is not comparable from image to image.

It is difficult to systematically assess whether a fluorescent protein will perturb the function of the protein it is fused to, because this perturbation depends on the molecular details of the interactions made by the protein. However, to partially assess the potential for perturbation of fusion protein function, we fused mTagBFP2 and the green and red fluorescent proteins to the septin Cdc12. We have previously observed that this protein is sensitive to C-terminal fusions. Fusions which disrupt the function of Cdc12 cause mislocalization of the protein, elongation of the yeast cell, or both. In Figure 4, we show images of yeast cells carrying each of these fusions. In general, the green fluorescent proteins perform well, with minimal effect on the localization of Cdc12. mTagBFP2 shows moderate perturbation to Cdc12 function, with some mislocalized Cdc12 and misshapen cells. The red fluorescent proteins have highly variable effects on Cdc12. In particular, a large fraction of Cdc12-mKO2 and Cdc12-mKate2 cells show mislocalized Cdc12, while fusions to the other red fluorescent proteins appear to function normally. This suggests that these two proteins may perturb other proteins as well. However, mKate2 has been successfully expressed in fusions to many mammalian proteins [21], so it may be worth trying in other protein fusions.

## Discussion

We have constructed a set of yeast optimized fluorescent protein tagging vectors expressing multiple blue, green, and red fluorescent proteins, as well as far-red and long-Stokes shift proteins. We have systematically expressed these as yeast fusions and assessed their brightness, photostability, and function as fusion proteins. We find that the blue fluorescent protein mTagBFP2, while ∼5-fold less detectable than EGFP, is bright enough to use as a third color in fluorescence microscopy. This is particularly useful for imaging with laser-based imaging systems with a 405 nm laser. The long-Stokes shift proteins and the far-red protein TagRFP657 are not bright enough to be useful tags. Somewhat surprisingly, we find that none of the green fluorescent proteins we have tested outperform EGFP. However we find a number of red fluorescent proteins that are brighter and more photostable than mCherry. The set of proteins we have constructed also includes the new Clover/mRuby2 FRET pair, which is an improved green/red replacement for CFP/YFP variants [16].

**Figure 5:**
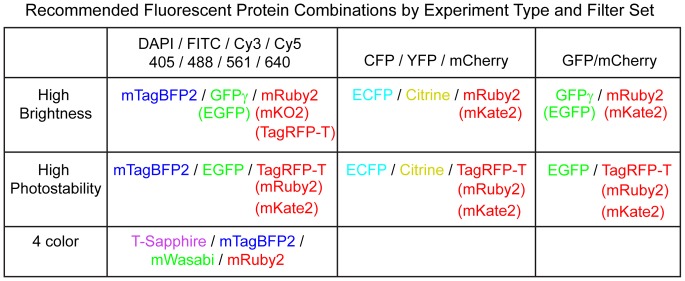
Recommended fluorescent protein combinations for yeast imaging. Recommended fluorescent protein combinations for imaging in yeast are broken down by filter set (horizontal axis) and experimental requirement (vertical axis). All proteins mentioned here are available in yeast tagging vectors either from this paper or from [6], [9] and most are available from Addgene. Recommended proteins are listed first, with alternatives given in parentheses. It is likely that iFP1.4 [22] or iRFP [23] can be used to image in the far-red (Cy5) channel, but this has not been tested in yeast. mWasabi is dimmer that EGFP or GFPγ, but is not excited at 405 nm, allowing it to be multiplexed with T-Sapphire [14].

Our recommendations for fluorescent proteins are summarized in Figure 5, broken down by the filter set used and the requirements of the experiment. When using imaging systems designed for DAPI/FITC/Cy3/Cy5, mTagBFP2, EGFP, and mRuby2 are an excellent set of proteins for three-color imaging. If an additional color is needed, it should be possible to multiplex T-Sapphire [14], mTagBFP2, mWasabi, and mRuby2; mWasabi is not excited at 405 nm, and T-Sapphire is a UV-excited, green emitting GFP variant that should not crosstalk with mTagBFP2 or mWasabi. When using a filter set with a Cy5 channel, it should be possible to image iFP1.4 [22]or iRFP [23] in the Cy5 channel, although these proteins have not been tested in yeast to our knowledge. The new red fluorescent proteins described here also offer improved options for imaging with CFP/YFP/RFP filter sets or GFP/mCherry filter sets. There are a number of improved CFP and YFP variants [24], [25] that may offer improved performance although these have not yet been tested in yeast. Additionally, the recently reported novel green fluorescent protein mNeonGreen [26] may be a brighter GFP replacement.

For experiments where photobleaching is not a concern, such as single time point imaging and flow cytometry, the green fluorescent protein of choice is either EGFP or GFPγ, which is ∼1.5× brighter. The choice of red fluorescent protein depends on the filter set used: for longer wavelength filter sets designed for mCherry, mKate2 (2.3× mCherry) is the brightest fluorescent protein. However, it perturbs Cdc12 when fused to it, so mRuby2 (1.7× mCherry and non-perturbative) may be preferred. For shorter wavelength filter sets designed for Cy3 or rhodamine, mKO2 (7.4× mCherry) is the brightest fluorescent protein. However, it also perturbs Cdc12 when fused to it, and mRuby2 is again the second-brightest protein (4.2× mCherry).

For experiments where photobleaching is a concern, such as time-lapse imaging, no green fluorescent protein outperforms EGFP. The most photostable red fluorescent protein is TagRFP-T, which we were able to collect 4.1× more light from than mCherry, before bleaching to 50% of its initial intensity. It is equally bright to mCherry in the mCherry channel and is brighter than mCherry in the Cy3 channel. It also appears to be non-perturbative in fusions. mKate2 and mRuby2 also outperform mCherry in photostability (2.6× and 1.5×, respectively).

Overall, for new tagging experiments, we recommend mTagBFP2 as the best blue fluorescent protein, EGFP as the best green fluorescent protein and TagRFP-T or mRuby2 as the best red fluorescent protein, depending on the requirements for photostability and brightness. mKate2 is also promising but fusions to it should be carefully assessed for perturbation. TagRFP-T and mRuby2 are also blue-shifted compared to mCherry and so perform better when used with shorter wavelength filters or 561 nm excitation. Together with EGFP and mTagBFP2, these provide a set of fluorescent proteins for three color imaging with 405 nm/488 nm/561 nm laser systems or common DAPI/FITC/Cy3 filter sets.

## Supporting Information

Table S1 **Photoactivatible/Photoconvertible proteins generated in this study.**
(DOCX)

Table S2 **Plasmids generated in this study.**
(DOCX)

Table S3 **Brightness and photostability of green fluorescent proteins.**
(DOCX)

Table S4 **Brightness of red fluorescent proteins.**
(DOCX)

Table S5 **Photostability of red fluorescent proteins.**
(DOCX)

References S1 **Supplementary references.**
(DOCX)

Sequences S1 **Protein sequences of the fluorescent proteins generated in this study.**
(DOCX)
