# Supplementary material for: Improved Blue, Green, and Red Fluorescent Protein Tagging Vectors for S. cerevisiae
Source: PLoS One. 2013 Jul 2;8(7):e67902. doi: 10.1371/journal.pone.0067902 (PMC3699464; doi:10.1371/journal.pone.0067902)
Supplement: Table S1 — Photoactivatible/Photoconvertible proteins generated in this study. (DOCX) [file pone.0067902.s001.docx]

Table S1: Photoactivatible / Photoconvertible proteins generated in this study.

| Protein | Reference |
| --- | --- |
| mEos2 | [1] |
| PA-mCherry | [2] |
| PA-TagRFP | [3] |
| PSmOrange | [4] |
| PS-CFP2 | [5] |
