# Supplementary material for: Improved Blue, Green, and Red Fluorescent Protein Tagging Vectors for S. cerevisiae
Source: PLoS One. 2013 Jul 2;8(7):e67902. doi: 10.1371/journal.pone.0067902 (PMC3699464; doi:10.1371/journal.pone.0067902)
Supplement: Table S2 — Plasmids generated in this study. (DOCX) [file pone.0067902.s002.docx]

Table S2: Plasmids generated in this study

| pFA6a-link-yomTagBFP-SpHis5 |
| --- |
| pFA6a-link-yomTagBFP2-SpHis5 |
| pFA6a-link-yoEGFP-SpHis5 |
| pFA6a-link-yoClover-SpHis5 |
| pFA6a-link-yoEmerald-SpHis5 |
| pFA6a-link-yoGFPγ-SpHis5 |
| pFA6a-link-yoMaxGFP-SpHis5 |
| pFA6a-link-yoSuperfolderGFP-SpHis5 |
| pFA6a-link-yomWasabi-SpHis5 |
| pFA6a-link-yomCherry-SpHis5 |
| pFA6a-link-yomApple-SpHis5 |
| pFA6a-link-yomKate2-SpHis5 |
| pFA6a-link-yomKO2-SpHis5 |
| pFA6a-link-yomRuby-SpHis5 |
| pFA6a-link-yomRuby2-SpHis5 |
| pFA6a-link-yoTagRFPT-SpHis5 |
| pFA6a-link-yoTagRFP657-SpHis5 |
| pFA6a-link-yomKeima-SpHis5 |
| pFA6a-link-yoLSSmKate2-SpHis5 |
| pFA6a-link-yomEos2-SpHis5 |
| pFA6a-link-yoPAmCherry-SpHis5 |
| pFA6a-link-yoPATagRFP-SpHis5 |
| pFA6a-link-yoPSmOrange-SpHis5 |
| pFA6a-link-yoPSCFP2-SpHis5 |
| pFA6a-link-yomTagBFP-CaUra3 |
| pFA6a-link-yomTagBFP2-CaUra3 |
| pFA6a-link-yoEGFP-CaUra3 |
| pFA6a-link-yoClover-CaUra3 |
| pFA6a-link-yoEmerald-CaUra3 |
| pFA6a-link-yoGFPγ-CaUra3 |
| pFA6a-link-yoMaxGFP-CaUra3 |
| pFA6a-link-yoSuperfolderGFP-CaUra3 |
| pFA6a-link-yomWasabi-CaUra3 |
| pFA6a-link-yomCherry-CaUra3 |
| pFA6a-link-yomApple-CaUra3 |
| pFA6a-link-yomKate2-CaUra3 |
| pFA6a-link-yomKO2-CaUra3 |
| pFA6a-link-yomRuby-CaUra3 |
| pFA6a-link-yomRuby2-CaUra3 |
| pFA6a-link-yoTagRFPT-CaUra3 |
| pFA6a-link-yoTagRFP657-CaUra3 |
| pFA6a-link-yomKeima-CaUra3 |
| pFA6a-link-yoLSSmKate2-CaUra3 |
| pFA6a-link-yomEos2-CaUra3 |
| pFA6a-link-yoPAmCherry-CaUra3 |
| pFA6a-link-yoPATagRFP-CaUra3 |
| pFA6a-link-yoPSmOrange-CaUra3 |
| pFA6a-link-yoPSCFP2-CaUra3 |
| pFA6a-link-yomTagBFP-KanR |
| pFA6a-link-yomTagBFP2-KanR |
| pFA6a-link-yoEGFP-KanR |
| pFA6a-link-yoClover-KanR |
| pFA6a-link-yoEmerald-KanR |
| pFA6a-link-yoGFP-KanR |
| pFA6a-link-yoMaxGFP-KanR |
| pFA6a-link-yoSuperfolderGFP-KanR |
| pFA6a-link-yomWasabi-KanR |
| pFA6a-link-yomCherry-KanR |
| pFA6a-link-yomApple-KanR |
| pFA6a-link-yomKate2-KanR |
| pFA6a-link-yomKO2-KanR |
| pFA6a-link-yomRuby-KanR |
| pFA6a-link-yomRuby2-KanR |
| pFA6a-link-yoTagRFPT-KanR |
| pFA6a-link-yoTagRFP657-KanR |
| pFA6a-link-yomKeima-KanR |
| pFA6a-link-yoLSSmKate2-KanR |
| pFA6a-link-yomEos2-KanR |
| pFA6a-link-yoPAmCherry-KanR |
| pFA6a-link-yoPATagRFP-KanR |
| pFA6a-link-yoPSmOrange-KanR |
| pFA6a-link-yoPSCFP2-KanR |
