# Supplementary material for: Improved Blue, Green, and Red Fluorescent Protein Tagging Vectors for S. cerevisiae
Source: PLoS One. 2013 Jul 2;8(7):e67902. doi: 10.1371/journal.pone.0067902 (PMC3699464; doi:10.1371/journal.pone.0067902)
Supplement: Table S3 — Brightness and photostability of green fluorescent proteins. (DOCX) [file pone.0067902.s003.docx]

Table S3: Brightness and photostability of green fluorescent proteins

|  | Widefield Brightness | | | Spinning Disk Brightness | | Photobleaching to 50% Initial Intensity | | | |
| --- | --- | --- | --- | --- | --- | --- | --- | --- | --- |
|  | Mean | SE | P | Mean | SE | Intensity | SE | Time | SE |
| EGFP | 1.00 | 0 |  | 1.00 | 0 | 1.00 | 0.00 | 1.00 | 0.00 |
| GFPγ | 1.55 | 0.09 | 0.01 | 1.67 | 0.37 | 0.40 | 0.05 | 0.23 | 0.03 |
| Clover | 1.13 | 0.07 | 0.07 | 1.06 | 0.23 | 0.21 | 0.03 | 0.22 | 0.04 |
| Emerald | 1.09 | 0.22 | 0.21 | 1.11 | 0.18 | 0.53 | 0.03 | 0.40 | 0.03 |
| MaxGFP | 0.81 | 0.12 | 0.76 | 0.75 | 0.16 | 0.35 | 0.02 | 0.57 | 0.02 |
| Superfolder GFP | 0.49 | 0.09 | 0.98 | 0.71 | 0.12 | 0.09 | 0.01 | 0.12 | 0.02 |
| Wasabi | 0.30 | 0.03 | 0.99 | 0.42 | 0.17 | 0.04 | 0.01 | 0.11 | 0.01 |

All values are measured relative to EGFP. SE is the standard error. P-values measure the likelihood that the true value is not greater than GFP. Photobleaching intensity is the mean integrated intensity recorded from the fluorescent protein during bleaching to 50% of its initial intensity. Photobleaching time is the mean time required for the protein to bleach to 50% of its initial intensity.
