# Supplementary material for: Improved Blue, Green, and Red Fluorescent Protein Tagging Vectors for S. cerevisiae
Source: PLoS One. 2013 Jul 2;8(7):e67902. doi: 10.1371/journal.pone.0067902 (PMC3699464; doi:10.1371/journal.pone.0067902)
Supplement: Table S4 — Brightness of red fluorescent proteins. (DOCX) [file pone.0067902.s004.docx]

Table S4: Brightness of red fluorescent proteins

|  | Widefield Brightness | | | | | | | |  | |
| --- | --- | --- | --- | --- | --- | --- | --- | --- | --- | --- |
|  | mCherry cube | | | Cy3 cube | | | Cy3 cube relative to mCherry | | Spinning Disk Brightness | |
| Protein | Mean | SE | P | Mean | SE | P | Mean | SE | Mean | SE |
| mCherry | 1.00 | 0 |  | 1.00 | 0 |  | 0.48 | 0.05 | 1.00 | 0 |
| mKate2 | 2.28 | 0.10 | 2.8e-5 | 1.61 | 0.09 | 0.003 | 0.66 | 0.15 | 2.22 | 0.15 |
| mRuby2 | 1.69 | 0.24 | 0.03 | 4.23 | 0.62 | 0.01 | 2.32 | 0.34 | 6.55 | 2.37 |
| mKO2 | 1.47 | 0.12 | 0.01 | 7.36 | 0.43 | 3.4e-4 | 3.07 | 0.73 | 7.37 | 1.55 |
| mApple | 1.31 | 0.10 | 0.02 | 2.18 | 0.23 | 0.01 | 0.93 | 0.25 | 4.65 | 0.73 |
| TagRFP-T | 1.20 | 0.21 | 0.20 | 2.99 | 0.62 | 0.03 | 1.29 | 0.46 | 6.18 | 0.93 |
| mRuby | 0.53 | 0.18 | 0.97 | 0.96 | 0.23 | 0.56 | 0.35 | 0.09 | 1.95 | 0.51 |

All values are measured relative to mCherry. SE is the standard error. P-values measure the likelihood that the true value is not greater than mCherry. Cy3 cube relative to mCherry compares the intensity of each protein measured with the Cy3 cube to the intensity of mCherry measured with the mCherry cube.
