# Supplementary material for: Improved Blue, Green, and Red Fluorescent Protein Tagging Vectors for S. cerevisiae
Source: PLoS One. 2013 Jul 2;8(7):e67902. doi: 10.1371/journal.pone.0067902 (PMC3699464; doi:10.1371/journal.pone.0067902)
Supplement: Table S5 — Photostability of red fluorescent proteins. (DOCX) [file pone.0067902.s005.docx]

Table S5: Photostability of red fluorescent proteins

|  | Intensity | | | Time | |
| --- | --- | --- | --- | --- | --- |
| Protein | Mean | SE | P | Mean | SE |
| mCherry | 1.00 | 0 |  | 1.00 | 0 |
| mKate2 | 2.58 | 0.46 | 0.02 | 1.12 | 0.11 |
| mRuby2 | 1.52 | 0.05 | 0.001 | 0.62 | 0.06 |
| mKO2 | 0.59 | 0.09 | 0.99 | 0.42 | 0.02 |
| mApple | 0.80 | 0.04 | 0.99 | 0.81 | 0.07 |
| TagRFP-T | 4.11 | 0.78 | 0.01 | 3.86 | 0.39 |
| mRuby | 0.49 | 0.15 | 0.98 | 0.86 | 0.05 |

All values are measured relative to mCherry. SE is the standard error. P-values measure the likelihood that the true value is not greater than mCherry. Intensity is the integrated intensity recorded from the fluorescent protein during bleaching to 50% of its initial intensity. Time is the time required for the protein to bleach to 50% of its initial intensity.
