# Supplementary material for: Improved Blue, Green, and Red Fluorescent Protein Tagging Vectors for S. cerevisiae
Source: PLoS One. 2013 Jul 2;8(7):e67902. doi: 10.1371/journal.pone.0067902 (PMC3699464; doi:10.1371/journal.pone.0067902)
Supplement: References S1 — Supplementary references. (DOCX) [file pone.0067902.s006.docx]

**Supplementary References:**

1. McKinney SA, Murphy CS, Hazelwood KL, Davidson MW, Looger LL (2009) A bright and photostable photoconvertible fluorescent protein. Nat Methods 6: 131–133. doi:10.1038/nmeth.1296.

2. Subach FV, Patterson GH, Manley S, Gillette JM, Lippincott-Schwartz J, et al. (2009) Photoactivatable mCherry for high-resolution two-color fluorescence microscopy. Nat Methods 6: 153–159. doi:10.1038/nmeth.1298.

3. Subach FV, Patterson GH, Renz M, Lippincott-Schwartz J, Verkhusha VV (2010) Bright monomeric photoactivatable red fluorescent protein for two-color super-resolution sptPALM of live cells. J Am Chem Soc 132: 6481–6491. doi:10.1021/ja100906g.

4. Subach OM, Patterson GH, Ting L-M, Wang Y, Condeelis JS, et al. (2011) A photoswitchable orange-to-far-red fluorescent protein, PSmOrange. Nat Methods 8: 771–777. doi:10.1038/nmeth.1664.

5. Chudakov DM, Verkhusha VV, Staroverov DB, Souslova EA, Lukyanov S, et al. (2004) Photoswitchable cyan fluorescent protein for protein tracking. Nat Biotechnol 22: 1435–1439. doi:10.1038/nbt1025.
