# Supplementary material for: Improved Blue, Green, and Red Fluorescent Protein Tagging Vectors for S. cerevisiae
Source: PLoS One. 2013 Jul 2;8(7):e67902. doi: 10.1371/journal.pone.0067902 (PMC3699464; doi:10.1371/journal.pone.0067902)
Supplement: Sequences S1 — Protein sequences of the fluorescent proteins generated in this study. (DOCX) [file pone.0067902.s007.docx]

mTagBFP

MSELIKENMH MKLYMEGTVD NHHFKCTSEG EGKPYEGTQT MRIKVVEGGP LPFAFDILAT SFLYGSKTFI

NHTQGIPDFF KQSFPEGFTW ERVTTYEDGG VLTATQDTSL QDGCLIYNVK IRGVNFTSNG PVMQKKTLGW

EAFTETLYPA DGGLEGRNDM ALKLVGGSHL IANIKTTYRS KKPAKNLKMP VYYVDYRLER KEANNETYVE

QHEVAVARYC DLPSKLGHKL N

mTagBFP2 (mTagBFP I174A)

MSELIKENMH MKLYMEGTVD NHHFKCTSEG EGKPYEGTQT MRIKVVEGGP LPFAFDILAT SFLYGSKTFI

NHTQGIPDFF KQSFPEGFTW ERVTTYEDGG VLTATQDTSL QDGCLIYNVK IRGVNFTSNG PVMQKKTLGW

EAFTETLYPA DGGLEGRNDM ALKLVGGSHL IANAKTTYRS KKPAKNLKMP VYYVDYRLER KEANNETYVE

QHEVAVARYC DLPSKLGHKL N

EGFP (GFP F64L S65T)

MSKGEELFTG VVPILVELDG DVNGHKFSVS GEGEGDATYG KLTLKFICTT GKLPVPWPTL VTTLTYGVQC FSRYPDHMKQ HDFFKSAMPE GYVQERTIFF KDDGNYKTRA EVKFEGDTLV NRIELKGIDF KEDGNILGHK LEYNYNSHNV YIMADKQKNG IKVNFKIRHN IEDGSVQLAD HYQQNTPIGD GPVLLPDNHY LSTQSALSKD PNEKRDHMVL LEFVTAAGIT LGMDELYK

Emerald (GFP F64L S65T S72A N149K M153T I167T)

MSKGEELFTG VVPILVELDG DVNGHKFSVS GEGEGDATYG KLTLKFICTT GKLPVPWPTL VTTLTYGVQC FARYPDHMKQ HDFFKSAMPE GYVQERTIFF KDDGNYKTRA EVKFEGDTLV NRIELKGIDF KEDGNILGHK LEYNYNSHKV YITADKQKNG IKVNFKTRHN IEDGSVQLAD HYQQNTPIGD GPVLLPDNHY LSTQSALSKD PNEKRDHMVL LEFVTAAGIT LGMDELYK

Clover (GFP 1-228 S30R Y39N S65G Q69A N105T Y145F M153T V163A I171V T203H)

MVSKGEELFT GVVPILVELD GDVNGHKFSV RGEGEGDATN GKLTLKFICT TGKLPVPWPT LVTTFGYGVA CFSRYPDHMK QHDFFKSAMP EGYVQERTIS FKDDGTYKTR AEVKFEGDTL VNRIELKGID FKEDGNILGH KLEYNFNSHN VYITADKQKN GIKANFKIRH NVEDGSVQLA DHYQQNTPIG DGPVLLPDNH YLSHQSALSK DPNEKRDHMV LLEFVTAA

Superfolder GFP (GFP S30R Y39N F64L S65T F99S N105T Y145F M153T V163A I171V A206V)

MSKGEELFTG VVPILVELDG DVNGHKFSVR GEGEGDATNG KLTLKFICTT GKLPVPWPTL VTTLTYGVQC FSRYPDHMKQ HDFFKSAMPE GYVQERTISF KDDGTYKTRA EVKFEGDTLV NRIELKGIDF KEDGNILGHK LEYNFNSHNV YITADKQKNG IKANFKIRHN VEDGSVQLAD HYQQNTPIGD GPVLLPDNHY LSTQSVLSKD PNEKRDHMVL LEFVTAAGIT LGMDELYK

GFPgamma (GFP F64L S65C S72A V163A I167T L231H)

MSKGEELFTG VVPILVELDG DVNGHKFSVS GEGEGDATYG KLTLKFICTT GKLPVPWPTL VTTFCYGVQC FARYPDHMKQ HDFFKSAMPE GYVQERTIFF KDDGNYKTRA EVKFEGDTLV NRIELKGIDF KEDGNILGHK LEYNYNSHNV YIMADKQKNG IKANFKTRHN IEDGSVQLAD HYQQNTPIGD GPVLLPDNHY LSTQSALSKD PNEKRDHMVL LEFVTAAGIT HGMDELYK

mWasabi

MVSKGEETTM GVIKPDMKIK LKMEGNVNGH AFVIEGEGEG KPYDGTNTIN LEVKEGAPLP FSYDILTTAF

SYGNRAFTKY PDDIPNYFKQ SFPEGYSWER TMTFEDKGIV KVKSDISMEE DSFIYEIHLK GENFPPNGPV

MQKETTGWDA STERMYVRDG VLKGDVKMKL LLEGGGHHRV DFKTIYRAKK AVKLPDYHFV DHRIEILNHD

KDYNKVTVYE IAVARNSTDG MDELYK

MaxGFP

MESDESGLPA MEIECRITGT LNGVEFELVG GGEGTPEQGR MTNKMKSTKG ALTFSPYLLS HVMGYGFYHF
GTYPSGYENP FLHAINNGGY TNTRIEKYED GGVLHVSFSY RYEAGRVIGD FKVMGTGFPE DSVIFTDKII
RSNATVEHLH PMGDNDLDGS FTRTFSLRDG GYYSSVVDSH MHFKSAIHPS ILQNGGPMFA FRRVEEDHSN
TELGIVEYQH AFKTPDADAG EE

mCherry

MVSKGEEDNM AIIKEFMRFK VHMEGSVNGH EFEIEGEGEG RPYEGTQTAK LKVTKGGPLP FAWDILSPQF MYGSKAYVKH PADIPDYLKL SFPEGFKWER VMNFEDGGVV TVTQDSSLQD GEFIYKVKLR GTNFPSDGPV MQKKTMGWEA SSERMYPEDG ALKGEIKQRL KLKDGGHYDA EVKTTYKAKK PVQLPGAYNV NIKLDITSHN EDYTIVEQYE RAEGRHSTGG MDELYK

TagRFP-T

MVSKGEELIK ENMHMKLYME GTVNNHHFKC TSEGEGKPYE GTQTMRIKVV EGGPLPFAFD ILATSFMYGS RTFINHTQGI PDFFKQSFPE GFTWERVTTY EDGGVLTATQ DTSLQDGCLI YNVKIRGVNF PSNGPVMQKK TLGWEANTEM LYPADGGLEG RTDMALKLVG GGHLICNFKT TYRSKKPAKN LKMPGVYYVD HRLERIKEAD KETYVEQHEV AVARYCDLPS KLGHKLNGMD ELYK

mRuby

MNSLIKENMR MKVVLEGSVN GHQFKCTGEG EGNPYMGTQT MRIKVIEGGP LPFAFDILAT SFMYGSRTFI

KYPKGIPDFF KQSFPEGFTW ERVTRYEDGG VITVMQDTSL EDGCLVYHAQ VRGVNFPSNG AVMQKKTKGW

EPNTEMMYPA DGGLRGYTHM ALKVDGGGHL SCSFVTTYRS KKTVGNIKMP GIHAVDHRLE RLEESDNEMF

VVQREHAVAK FAGLGGG

mRuby2 (mRuby L15M I102V A119V A131P)

MVSKGEELIK ENMRMKVVME GSVNGHQFKC TGEGEGNPYM GTQTMRIKVI EGGPLPFAFD ILATSFMYGS RTFIKYPKGI PDFFKQSFPE GFTWERVTRY EDGGVVTVMQ DTSLEDGCLV YHVQVRGVNF PSNGPVMQKK TKGWEPNTEM MYPADGGLRG YTHMALKVDG GGHLSCSFVT TYRSKKTVGN IKMPGIHAVD HRLERLEESD NEMFVVQREH AVAKFAGLGG GMDELYK

mApple

MVSKGEENNM AIIKEFMRFK VHMEGSVNGH EFEIEGEGEG RPYEAFQTAK LKVTKGGPLP FAWDILSPQF

MYGSKVYIKH PADIPDYFKL SFPEGFRWER VMNFEDGGII HVNQDSSLQD GVFIYKVKLR GTNFPSDGPV

MQKKTMGWEA SEERMYPEDG ALKSEIKKRL KLKDGGHYAA EVKTTYKAKK PVQLPGAYIV DIKLDIVSHN

EDYTIVEQYE RAEGRHSTGG MDELYK

mKO2

MVSVIKPEMK MRYYMDGSVN GHEFTIEGEG TGRPYEGHQE MTLRVTMAEG GPMPFAFDLV SHVFCYGHRV

FTKYPEEIPD YFKQAFPEGL SWERSLEFED GGSASVSAHI SLRGNTFYHK SKFTGVNFPA DGPIMQNQSV

DWEPSTEKIT ASDGVLKGDV TMYLKLEGGG NHKCQMKTTY KAAKEILEMP GDHYIGHRLV RKTEGNITEQ

VEDAVAHS

mKate2

MVSELIKENM HMKLYMEGTV NNHHFKCTSE GEGKPYEGTQ TMRIKAVEGG PLPFAFDILA TSFMYGSKTF

INHTQGIPDF FKQSFPEGFT WERVTTYEDG GVLTATQDTS LQDGCLIYNV KIRGVNFPSN GPVMQKKTLG

WEASTETLYP ADGGLEGRAD MALKLVGGGH LICNLKTTYR SKKPAKNLKM PGVYYVDRRL ERIKEADKET

YVEQHEVAVA RYCDLPSKLG HR

TagRFP657

MSELITENMH MKLYMEGTVN NHHFKCTSEG EGKPYEGTQT QRIKVVEGGP LPFAFDILAT SFMYGSHTFI

NHTQGIPDFW KQSFPEGFTW ERVTTYEDGG VLTATQDTSL QDGCLIYNVK IRGVNFPSNG PVMQKKTLGW

EAHTEMLYPA DGGLEGRTAL ALKLVGGGHL ICNFKTTYRS KKPAKNLKMP GVYYVDYRLE RIKEADKETY

VEQHEVAVAR YCDLPSKLGH KLN

mKeima

MVSVIAKQMT YKVYMSGTVN GHYFEVEGDG KGKPYEGEQT VKLTVTKGGP LPFAWDILSP QLQYGSIPFT

KYPEDIPDYF KQSFPEGYTW ERSMNFEDGA VCTVSNDSSI QGNCFIYNVK ISGENFPPNG PVMQKKTQGW

EPSTERLFAR DGMLIGNDYM ALKLEGGGHY LCEFKSTYKA KKPVRMPGRH EIDRKLDVTS HNRDYTSVEQ

CEIAIARHSL LG

LSS-mKate2

MVSELIKENM HMKLYMEGTV NNHHFKCTSE GEGKPYEGTQ TMRIKVVEGG PLPFAFDILA TSFMYGSYTF

INHTQGIPDF FKQSFPEGFT WERVTTYEDG GVLTATQDTS LQDGCLIYNV KIRGVNFTSN GPVMQKKTLG

WEAGTEMLYP ADGGLEGRSD DALKLVGGGH LICNLKSTYR SKKPAKNLKV PGVYYVDRRL ERIKEADKET

YVEQHEVAVA RYCDLPSKLG HR

mEos2

MSAIKPDMKI KLRMEGNVNG HHFVIDGDGT GKPFEGKQSM DLEVKEGGPL PFAFDILTTA FHYGNRVFAK

YPDNIQDYFK QSFPKGYSWE RSLTFEDGGI CIARNDITME GDTFYNKVRF YGTNFPANGP VMQKKTLKWE

PSTEKMYVRD GVLTGDIHMA LLLEGNAHYR CDFRTTYKAK EKGVKLPGYH FVDHCIEILS HDKDYNKVKL

YEHAVAHSGL PDNARR

PA-TagRFP

MVSKGEELIK ENMHMKLYME GTVNNHHFKC TSEGEGKPYE GTQTMRIKVV EGGPLPFAFD ILATSFMYGS STFINHTQGI PDFWKQSFPE GFTWERVTTY EDGGVLTATQ DTSLQDGCLI YNVKIRGVNF PSNGPVMKKK TLGWEPSTEK LKPADGGLEG RVDMALKLVG GGHLICNFKT TYRSKKPAKN LKMPGVYYVD RRLEIIKEAD KETYWEQHEV AVARYSDLPS KLGHR

PS-CFP2

MSKGAELFTG IVPILIELNG DVNGHKFSVS GEGEGDATYG KLTLKFICTT GKLPVPWPTL VATLSYGVQC FSRYPDHMKQ HDFFKSAMPE GYIQERTIFF EDDGNYKTRA EVKFEGDTLV SRIELTGTDF KEDGNILGNK MEYNYNATNV YIVADKARNG IKVNFKVRHN IKDGSVQLAD HYQQNTPIGD GPVLLPDNHY LSTQSALSKD PNEKRDHMIY LEFVTAAAIT HGMDELYK

PA-mCherry

MVSKGEEDNM AIIKEFMRFK VHMEGSVNGH VFEIEGEGEG RPYEGTQTAK LKVTKGGPLP FTWDILSPQF MYGSNAYVKH PADIPDYFKL SFPEGFKWER VMKFEDGGVV TVTQDSSLQD GEFIYKVKLR GTNFPSDGPV MQKKTMGWEA LSERMYPEDG ALKGEVKPRV KLKDGGHYDA EVKTTYKAKK PVQLPGAYNV NRKLDITSHN EDYTIVEQYE RAEGRHSTGG MDELYK

PSmOrange

MVSKGEENNM AIIKEFMRFK VRMEGTVNGH EFEIEGEGEG RPYEGFQTAK LKVTKGGPLP FAWDILSPLF TYGSKAYVKH PADIPDYFKL SFPEGFKWER VMNYEDGGVV TVTQDSSLQD GEFIYKVKMR GTNFPSDGPV MQKKTMGWEA SSERMYPEDG ALKGEIRMRL KLKDGGHYTS EVKTTYKAKK SVQLPGAYIV GIKLDITSHN EDYTIVEQYE RAEGRHSTGG MDELYK
